# Antibody-Based Immunotherapy: Alternative Approaches for the Treatment of Metastatic Melanoma

**DOI:** 10.3390/biomedicines8090327

**Published:** 2020-09-03

**Authors:** Fleury Augustin Nsole Biteghe, Nyangone Ekome Toung Chalomie, Neelakshi Mungra, Guillaume Vignaux, Nan Gao, Aurelia Vergeade, Ambrose Okem, Krupa Naran, Jean De La Croix Ndong, Stefan Barth

**Affiliations:** 1 Department of Radiation Oncology and Biomedical Sciences, Cedars-Sinai Medical, 8700 Beverly Blvd, Los Angeles, CA 90048, USA; fleury.nsolebiteghe@cshs.org; 2 Zhongshan Medical School, Sun Yat-Sen University, Guangzhou 510275, China; shalomyobame@gmail.com; 3 Medical Biotechnology & Immunotherapy Research Unit, Institute of Infectious Disease and Molecular Medicine, Faculty of Health Sciences, University of Cape Town, Cape Town 7700, South Africa; mngnee002@myuct.ac.za (N.M.); krupa.naran@uct.ac.za (K.N.); 4 Arctic Slope Regional Corporation (ASRC) Federal, Beltsville, MD 20705, USA; guillaume.vignaux@gmail.com; 5 School of Arts and Science, Rutgers University, New Brunswick, NJ 08901, USA; ngao@newark.rutgers.edu (N.G.); jn568@newark.rutgers.edu (J.D.L.C.N.); 6 Department of Clinics and Pharmacology, Vanderbilt Medical Center, Nashville, TN 37232, USA; aurelia.vergeade@gmail.com; 7 Department of Anaesthesia, School of Clinical Medicine, Faculty of Health Sciences, University of Witwatersrand, Johannesburg 2193, South Africa; aokem39@gmail.com; 8 Department of Orthopedic Surgery, School of Medicine, New York University, New York, NY 10003, USA; 9 Cancer Biotechnology, Department of Integrative Biomedical Sciences, Faculty of Health Sciences, University of Cape Town, Cape Town 7700, South Africa

**Keywords:** antibody–drug conjugates (ADCs), skin cancer (melanoma), photodynamic therapy (PDT), photoimmunotherapy (PIT)

## Abstract

Melanoma is the least common form of skin cancer and is associated with the highest mortality. Where melanoma is mostly unresponsive to conventional therapies (e.g., chemotherapy), BRAF inhibitor treatment has shown improved therapeutic outcomes. Photodynamic therapy (PDT) relies on a light-activated compound to produce death-inducing amounts of reactive oxygen species (ROS). Their capacity to selectively accumulate in tumor cells has been confirmed in melanoma treatment with some encouraging results. However, this treatment approach has not reached clinical fruition for melanoma due to major limitations associated with the development of resistance and subsequent side effects. These adverse effects might be bypassed by immunotherapy in the form of antibody–drug conjugates (ADCs) relying on the ability of monoclonal antibodies (mAbs) to target specific tumor-associated antigens (TAAs) and to be used as carriers to specifically deliver cytotoxic warheads into corresponding tumor cells. Of late, the continued refinement of ADC therapeutic efficacy has given rise to photoimmunotherapy (PIT) (a light-sensitive compound conjugated to mAbs), which by virtue of requiring light activation only exerts its toxic effect on light-irradiated cells. As such, this review aims to highlight the potential clinical benefits of various armed antibody-based immunotherapies, including PDT, as alternative approaches for the treatment of metastatic melanoma.

## 1. Introduction

Melanoma represents the most aggressive, malignant phenotype resulting from a genetic and/or environmental-induced change to epidermal skin melanocytes and accounts for more than 75% of skin cancer-related deaths [1,2]. It mostly affects light-skinned individuals who are excessively exposed to solar ultraviolet radiation A and B (UVA and UVB), which are able to indirectly or directly cause DNA damage through oxidative (reactive oxygen species, ROS) or genotoxic stresses, respectively [3,4,5]. Alternatively, a genetic predisposition acquired through B-Raf proto-oncogene (BRAF^V600E^) mutation (present in more than 60% of melanoma patients) is characterized by the substitution of the amino acid aspartic acid by valine at position 600 and may lead to melanoma pathogenesis or melanomagenesis [6]. BRAF^V600E^ induces constitutive kinase activity (e.g., mitogen-activated protein kinase pathway activation known as MAPK) which drives the uncontrolled growth of melanoma cells and pro-tumorigenic angiogenesis leading to disease metastases [6,7].

To date, the gold standard of therapy for malignant melanoma tumor is surgical resection [2]. Once melanomas reach the advanced metastatic stage, systemic therapies using the first US Food and Drug Administration (FDA)-approved chemotherapeutic drug dacarbazine (DTIC) and high-dose interleukin-2 (HD-IL-2, FDA-approved in 1998) have become the mainstay of treatments [8,9,10,11,12,13,14]. Unfortunately, the clinical success of these systemic therapies was hampered by severe dose-limiting toxicities, which did not improve overall patient survival [14,15]. In light of this, novel palliative treatment approaches were urgently needed to specifically treat patients with refractory and metastatic disease and to help circumvent these undesired toxicities to improve the overall therapeutic efficacy and patient survival. Thus far, two targeted therapeutic approaches have been developed using antagonist monoclonal antibodies (mAbs) or small molecules such as vemurafenib (FDA-approved, 2011), dabrafenib (FDA-approved, 2013), trametinib (FDA-approved, 2013), encorafenib, and binimetinib (FDA-approved, 2018) to obstruct agonistic ligand binding to cognate overexpressed tumor-associated antigen receptors (TAAs) or by inhibiting the oncogenic BRAF/MAPK/MEK (MEK: mitogen-activated protein kinase kinase) (originally known as extracellular signal-regulated kinases)-signaling axis [6,16,17,18]. However, the compromised efficacy of vemurafenib and other BRAF/MEK inhibitors have been associated with aberrant expression of membrane proteins known as ATP-binding cassette (ABC) transporters (e.g., ABCB5 and ABCG2), mediating cellular resistance by extruding cytotoxic molecules out from cells, as well as the re-activation of the MAPK pathway and to a lesser extent phosphatidylinositol-3 kinase (PI3K)–protein kinase-B (Akt) pathway activation and phosphatase and tensin homolog (PTEN) loss following prolonged targeted therapy treatments [6,19,20,21,22,23]. In contrast to BRAF inhibitors, mAbs partly exert their cytotoxic effects by reducing ectodomain density or by inducing receptor-mediated endocytosis through the activation of antibody-dependent cellular cytotoxicity (ADCC) toward tumor cells overexpressing the specific TAA [19,20,21]. In 2011 and 2014, ipilimumab (anti-cytotoxic T-lymphocyte antigen-4 (CTLA-4)) and pembrolizumab (anti-programmed cell death-1 (anti-PD-1)) were FDA-approved respectively, to treat melanoma patients with disseminated tumor, and they produced better patient survival (from 9 months to over 3.5 years), which was mediated through an antitumor immune response [6,20,24,25,26]. Despite obvious clinical benefits, these immunotherapies were associated with undesirable side effects, partly owing to the bulky size of mAbs, which limited tumor penetration, as well as the rodent origin of the mAb, hence inducing an immune response when administered to immunocompetent patients [26,27].

To mitigate these undesired effects, antibody–drug conjugates (ADCs) consisting of a toxic molecule conjugated to a mAb were developed and served as a vehicle to specifically deliver toxic molecules within cancer cells expressing the cognate TAA [27,28,29,30]. To achieve their therapeutic goals, ADCs exert cytotoxicity by (1) exploiting the differential expression of TAAs between targeted and healthy tissues, and (2) by using the mAb as a vehicle to specifically transport the conjugated toxic warhead to the diseased site [29,31,32]. So far, ADCs have been able to improve the toxicity/therapy balance paradigm in multiple pre-clinical models of solid melanoma tumors overexpressing epidermal growth factor receptor (EGFR), chondroitin sulfate proteoglycan 4 (CSPG4), pigment-forming protein (PMEL17), and melanotransferrin (p97) [16,30,33,34]. The best ADCs would ideally be non-toxic in their native administered state, with toxicity only unleashed when internalized in targeted tumor cells. With the recent biotechnological advances, a novel approach using this principle was developed in the form of targeted photodynamic therapy (PDT), using a death-inducing amount of reactive oxygen species (ROS) to induce tumor destruction [17,35]. This targeted PDT also known as photoimmunotherapy (PIT) has shown therapeutic benefit in comparison to standard ADCs, in necessitating an extra step of light activation to unleash phototoxicity [35,36,37,38,39,40,41,42].

### 1.1. Summary of FDA-Approved Melanoma Chemotherapy and Targeted Therapies

**Figure 1:**
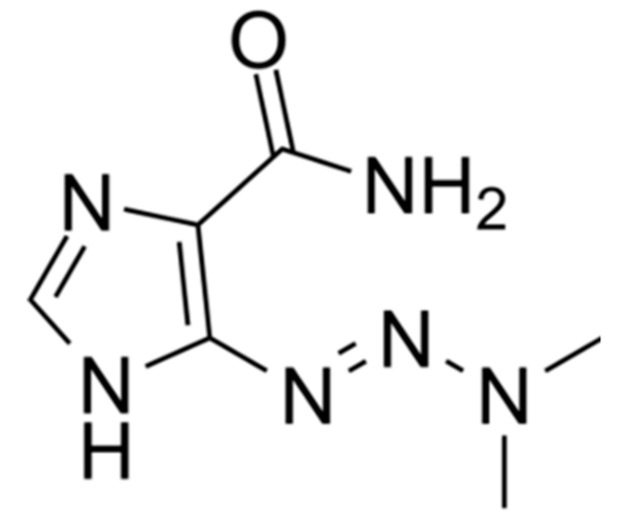
The chemical structure of dacarbazine.

For many years until 2011, FDA-approved DTIC (Figure 1) [14,43] has been used as the mainstay of treatment of metastatic melanoma patients with unresectable tumors. DTIC is an alkylating agent, which is administered as a prodrug, 5-[3methyltriazen-lyl]-imidazole-4-carboxamide (MTIC), which exerts its cytotoxic effect by forming methyl-DNA adducts, activating cell cycle arrest and cell death [10,44,45]. DTIC remains the best chemotherapeutic agent to treat metastatic melanoma, with the best overall (15–25%) and complete treatment responses (5%) [4,13,44,46]. Yet, it has not been successful in improving patient survival [13,47,48]. DTIC is mostly used in the clinic in doses ranging from 150 to 200 mg/m^2^ for a period of 5 days [13]. However, DTIC 200 mg/m^2^ treatment was shown to be more suitable and well-tolerated by patients if given in a single dose of 800 to 1000 mg/m^2^ per week and repeated every 3 to 4 weeks, based on reduced toxicity [13]. In comparison to other combinatorial approaches with temozolomide, cisplatin, tamoxifen, and vinblastine, DTIC monotherapy has demonstrated a significant improvement of patient outcomes [13,14,49].

However, clinical approval of orally administrated vemurafenib (FDA-approved, 2011: 960 mg twice daily) and dabrafenib (FDA-approved, 2013: 150 mg twice daily) was achieved based on their abilities to significantly improve both overall survival (13.6 and 20 months) and progression-free survival (5.3 and 6.9 months) in BRAF^V600E^-treated metastatic melanoma patients when compared to DTIC treated control offering poorer overall (9.7 and 15.6 months) and progression-free survival (1.6 and 2.7 months), respectively [6]. Nevertheless, the clinical benefits of the aforementioned targeted therapies were short-lived due to the development of resistance [6,22,23,50]. Recently, Erdmann et al. (2020) have shown that prolonged exposure (12 months) of BRAF^V600E^-positive human-derived melanoma cells to vemurafenib simultaneously desensitized them to both vemurafenib and DTIC treatments [22]. The mechanism underlying the latter resistance was a combination of MAPK pathway re-activation (MAPK is originally inactivated by BRAF inhibitors such as vemurafenib), MEK and PI3K/Akt activities, driving DTIC-acquired cross-resistance [6,22,23]. Collectively, these findings corroborated with previous reports illustrating the association of PTEN (PTEN: tumor suppressor and PI3K antagonist) with PI3K/Akt activation and resistance to BRAF inhibitors [21]. Additionally, clinical reports have revealed that patients with PTEN loss and MEK activation have shorter progression-free survival following dabrafenib treatments [6,51,52]. Therefore, efforts to mitigate the MEK-induced BRAF-resistance were developed and led to the FDA approval (in 2013) of trametinib (a MEK inhibitor) [53]. Clinically, orally administrated trametinib (2 mg daily) prolonged progression-free survival (4.9 versus 1.6 months of DTIC and paclitaxel treatments) and overall survival up to 6 months in 81% of BRAF^V600E^-positive treated metastatic melanoma patients compared to 61% among the DTIC or paclitaxel-treated controls [6,53]. Furthermore, the FDA recently approved (2018) the clinical usage of the combined therapy using encorafenib and binimetinib for treating melanoma patients bearing BRAF^V600E^ mutations, due to superior progression-free survival (14.9 months) on the vemurafenib-treated group (7.3 months) [18].

Based on the therapeutic success of targeted therapies, another combinatorial treatment approach was developed by Ryabaya et al. (2017) through a combination of temozolomide (DTIC analog) with an autophagy inhibitor (hydroxychloroquine or chloroquine LY294002) [54,55]. These therapeutic regimens were shown to significantly enhance the therapeutic efficacy in melanoma patients [54,55]. Therefore, it was hypothesized that new adjunctive therapeutic approaches combining chemotherapy with light-sensitive PDT could further ameliorate therapeutic efficacy and reduce side effects as reported in many cancers such as breast, ovarian, melanoma and non-melanoma cancers [8,44,45,46,56,57,58,59].

### 1.2. Photodynamic Therapy Treatment

#### 1.2.1. Mechanism of Action

**Figure 2:**
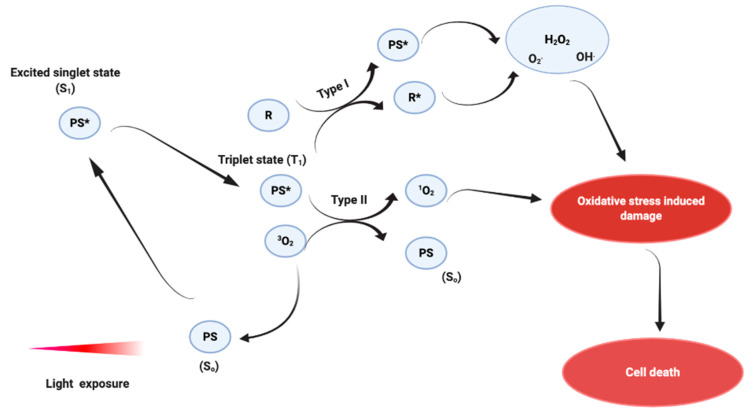
The “trinity” (photosensitizer, oxygen, and light) of photodynamic therapy (PDT). The photosensitizer (PS) in its stable state (S_o_) absorbs the photon of an appropriate monochromatic light source to form an excited singlet state (S_1_). Thereafter, S_1_ can transfer energy to an excited triplet (T_1_) and then either forms radicals via Type I reactions or transfers its energy in the form of electrons to molecular oxygen and forms singlet oxygen (^1^O_2_) species through a Type II reaction, which oxidatively damages tumor cells. Note: arrows indicate the transfer/change from one state to another.

Although PDT has not yet been approved by the FDA for melanoma treatment, it is a well-known and established therapeutic approach currently used to treat various types of cancers such as bladder, basal cell carcinoma, and some non-melanoma skin cancers (for e.g., Actinic keratosis and Bowen’s disease) [47,49,60,61,62,63]. Recently, combination therapy using PDT (using Redaporfin photosensitizer (PS)) and nivolumab (anti-PD-1) was shown to clinically eradicate all visible tumors on a head and neck cancer patient, who previously relapsed from surgery, radiotherapy, and various systemic treatments [64]. Likewise, PDT using SGX301 (Synthetic Hypericin) is presently showing promising results in a phase III clinical trial (ClinicalTrials.gov Identifier: NCT02448381) in the treatment of cutaneous T-cell lymphoma (CTCL). The delay in approval of PDT for melanoma treatment could be due to multiple factors such as the elevated antioxidant status and the light-shielding effects of melanin [48,58,65]. PDT is a form of minimally invasive therapeutic strategy exerting a selective phototoxic activity only toward irradiated cancer cells in the presence of a light-sensitive PS molecule, a visible light source, and oxygen [2,66]. Upon light activation at a specific wavelength, the PS is raised from an unexcited ground state (S_0_) to a very unstable excited triplet state (S_1_) by absorbing energy in the form of photons. In its triplet state, the PS is unstable and can decay back to the ground state through energy conversion either into heat or fluorescence, which can be used for the purposes of photodiagnosis [66,67,68]. In addition, S_1_ molecules can react with an electron-donating substrate to generate PSs that eventually react with oxygen to form superoxide anion (O_2_^−^) and hydroxyl radicals (OH^.^), thus producing ROS in a type I photochemical reaction (Figure 2) [2,66,68]. Alternatively, S_1_ can via a type II photochemical reaction transfer electrons to ground-state oxygen and form singlet oxygens (^1^O_2_) (Figure 2) [2,66,68]. These oxidative damages are short-lived and exert their phototoxic effects in a very small radius (20 nm) by destroying tumor vasculature, thereby activating anti-tumor immune responses and cell death mechanisms [2,49,69]. It has been demonstrated that the PS subcellular localization plays an important role in dictating the type of cell death mechanism which should be activated to cause tumor destruction [2,70,71,72,73].

#### 1.2.2. Hypericin

The success of PDT is related to 3 factors: (1) the PS, (2) the presence of oxygen in the immediate environment, and (3) the induction of ROS through an appropriate photoactivation at a specific wavelength of light. Hypericin is a natural PS that is biosynthesized within the dark glands of the petals and leaves of the St John’s Wort plant (*Hypericum perforatum*) [74,75]. It belongs to the chemical class of naphthodianthrones and can be chemically synthesized through the conversion of emodin to hypericin by the Hyp-1 enzyme, yielding approximately 84.6% of efficient conversion of hypericin when overexpressed in *E. coli* [76,77]. This is a favorable alternative, as direct extraction from *H. perforatum* produces a low yield of hypericin due to the low occurrence of the naphthodianthrones (0.05–0.3%), which are costly and necessitate multiple cycles of purification, while requiring fast handling of materials [77,78]. Hypericin absorbs at both the 300–400 nm (ultraviolet) and 500–600 nm (white light) range, with an optimal absorption peak at 563 nm and an emission at 600 nm [58,79]. In humans, hypericin has been used for the treatment of various conditions including depression, anxiety, restlessness, and sleeping disorders [80,81]. Moreover, its fluorescent properties have enabled the visualization of malignant tumors such as gliomas in a process known as photodiagnosis [82,83,84]. In addition, its photosensitizing properties have been widely used in treating several cancers (GH4C1 rat adenoma, human P3 squamous carcinoma cells, head and neck cancer, and melanoma) [8,85,86,87,88]. Hypericin has desirable properties as a PS, since it has no dark cytotoxicity, low photobleaching, an intense absorption spectrum in the visible light region, a large excitation range, and it is rapidly cleared from the body while being preferentially retained within the tumor [84,89,90,91,92]. Due to its hydrophobic nature, hypericin is mainly internalized within tumor cells through passive diffusion or by forming a complex with the low-density lipoproteins (LDL), which is overexpressed in the majority of cancer cells [75,91,93]. During PDT, hypericin mainly exercises its cytotoxic effect through the production of singlet oxygens (^1^O_2_) [94,95], superoxide anion along with other ROS. Moreover, the production of ROS by hypericin has been shown to induce cell death through mechanisms such as apoptosis, necrosis, and autophagy [78,89,96,97,98,99]. This has been reported to be associated with hypericin-specific subcellular localization within the cells after PDT treatment [70,71,78,100].

#### 1.2.3. Photodynamic Therapy and Melanoma

PDT is a localized therapy (by virtue of the tumor-specific irradiation) that is minimally toxic to healthy tissues and rarely induces therapeutic resistance [101,102]. Clinically, PDT was able to completely eradicate basal cell carcinoma (BCC) tumors in 95.4% of patients treated with methyl aminolevulinate (MAL) [44]. PDT has been investigated in vitro and in vivo as a potential adjuvant therapy with promising outcomes for the treatment of metastatic melanoma [2,103]. A study by Sheleg et al. (2004) reported the complete remission of patients treated with a double exposure to chlorin e6 (Ce6) and PDT, with no recurrence [104]. This prompted further investigations aiming to achieve clinical success of PDT for melanoma as a treatment option. In melanoma, PDT has shown to mostly cause tumor destruction via apoptosis [78,105]. As such, the PS photofrin was able to induce apoptotic cell death (in 90% of melanoma cells) dependent on the PS concentration and exposure time [105]. This was further corroborated by Robertson et al. (2010) and Li et al. (2018), who showed the antiproliferative apoptotic-inducing PDT effects of 5-aminolevulinic-acid (5-ALA), methylene blue, and metallophthalocyanin on melanoma cells [106]. Interestingly, this apoptotic cell death induction was shown to correlate with PS mitochondrial subcellular localization as demonstrated in a study by Choramanska et al. (2012), in which the subcellular accumulation of photofrin in the mitochondrial membrane of Mel5 cells was associated with mitochondrial membrane disruption and apoptosis [107]. This finding was further illustrated in another study by Kleemann et al. (2014), showing that hypericin could co-localize within the endoplasmic reticulum (ER), lysosomes, mitochondria, and melanosomes for about 4 hours post-incubation [78]. Apoptosis was induced in pigmented and unpigmented melanomas in a caspase-dependent manner and in moderated pigmented melanomas in a caspase-independent manner [78]. Additionally, hypericin-based PDT (henceforth referred as HYP-PDT) treatment induced cell death differentially depending on the cell types, pigmentation, and PS cellular localization [78,100]. On the other hand, necrotic cell deaths were observed in melanocytes and pigmented melanoma, whereas an apoptotic-like programmed cell death was predominantly observed in HYP-PDT treated keratinocytes and unpigmented melanoma [100]. Overall, it was deduced that this HYP-PDT induced necrosis by ROS-dependent mechanisms, which result in an increase in melanosome membranes permeability, causing melanogenesis by-products leakage into the cytoplasm [100]. Conversely, the initiation of keratinocytes and unpigmented melanoma apoptosis was associated with a disturbance of the functions of both the ER and mitochondria, which led to cell surface translocation of damage-associated molecular patterns (DAMPs) such as calreticulin (CRT), thus stimulating an apoptotic immune-mediated cell death [78,100,108,109,110]. However, no considerable changes were detected in the melanosome-like lysosomes. This observation may possibly identify an intrinsic cellular resistance mechanism to PDT strategy and hence an associate protective role of melanin and melanosomes in PDT and chemotherapy [48,111].

The depigmentation of re-sensitized melanoma cells previously resistant to PDT supports the involvement of melanin in lessening PDT efficacy [48]. This was confirmed in a pre-clinical study, where pigmented melanoma was found to be less responsive to PDT than their unpigmented counterpart [65]. Therefore, efforts to bypass this resistance led to the successful combination treatment strategy of melanoma and glioblastoma cancers by applying a chemotherapeutic agent such as DTIC or an analogue (temozolomide) with HYP-PDT [8,111]. The rationale behind these therapeutic approaches was to induce an oxidative (induced by HYP-PDT) and a genotoxic stress (induced by DTIC or temozolomide) to overcome chemoresistance, thus inducing cell death. This combination therapy (DTIC+HYP-PDT) could synergistically kill human glioblastoma and melanoma cells when compared to their respective monotherapies [8,112]. DTIC combined with HYP-PDT not only improved the efficacy of the treatment but also offered a possible reduction in chemotherapeutic doses, potentially reducing the off-target effects of the respective monotherapies. Recently, Theodossiou et al. (2019) reported that combination therapy using HYP-PDT and tamoxifen could effectively kill breast cancer cells with over 90% cytotoxicity through necrosis and autophagy (controlling the turnover of organelles and proteins within cells, and of cells within organisms) [113]. Likewise, Lin et al. (2017) showed that HYP-PDT could re-sensitize previously oxaliplatin-resistant colon cancer cells through autophagic cell death mode activation [114]. Similarly, recent studies have demonstrated that sinoporphyrin sodium (DVDMS) could induce cell death on esophageal cancer cells, through autophagy, which inhibited apoptosis [115,116]. In contrast, Kaizhen et al. (2018) showed that autophagy has a pro-survival role, which is abrogated through a dual action of 3-methyladenine (autophagic inhibitor) and Ce6-PDT successfully inducing apoptosis [116]. Currently, the therapeutic efficacy of HYP-PDT is being tested in a phase III clinical trial (NCT02448381) for the treatment of cutaneous T-cell lymphoma. Of late, Sun et al. (2017) demonstrated how rod-shaped gold nanoparticles (AuNRs) and Ce6-doped mesoporous silica nanorods would allow synergistically combining AuNR-induced photothermal therapy (PTT) (increasing tumor temperature above body temperatures of 42–45 °C) and Ce6-activated PDT as demonstrated in pre-clinical animal models [115]. This latter combined therapy could enable the monitoring of the therapeutic responses using photoacoustic (AuNR) or near-infrared (NIR) imaging (using Ce6) respectively [117]. Likewise, Wu et al. (2017) revealed the theragnostic properties of a graphene–Au–PEG/Ce6 (GO/AuNS–PEG/Ce6) nanohybrid system, in eradicating EMT6 xenograft tumors through PTT and PDT synergism, using a single NIR laser irradiation (660 nm) [118]. According to the authors, the success of this therapeutic modality was based on the specific subcellular localization of GO/AuNS–PEG/Ce6 within the mitochondrial and lysosomal membranes, which were photodamaged to induce cell death, hence tumor destruction [118]. These results were corroborated by Yan et al. (2018), showing how Ce6-PDT in combination with AuNRs could collaboratively eradicate tumors in mice 16 days post therapy [119]. With this in mind, Xu et al. (2019) developed a nanorod platform that was functionalized to carry AuNRs, 5-aminolevulinic acid (5-ALA)–PS and antibodies targeting CD44 and the human epidermal growth factor receptor-2 (HER-2) overexpressing breast cancers [120]. During this study, Xu et al. were able to specifically deliver this nanoplatform in CD44 and HER-2 expressing MCF-7 cancer cells, which concurrently allowed the destruction of tumors through a ROS and thermal-mediated cell death [120]. Based on these therapeutic successes, pilot clinical trials were initiated to investigate the theranostic (NCT02680535) and therapeutic effects of Au nanoparticle mediated-PDT (NCT00848042) in prostate and head and neck cancer patients respectively [121]. Collectively, these combination therapies clearly present benefits over monotherapies. However, their long-lasting therapeutic effects should be assessed to avoid relapses. Remarkably, it has been shown in melanomas that the trapping properties of anticancer drugs by melanosomes could be attributed to the ABC transporter proteins, which are located and expressed in the membrane of melanosomes and other organelles, where they neutralize toxic substances by internalizing them into the melanosomes or by extruding them out of the cells to diminish treatment efficacy [2,111]. Hence, this subcellular localization and preservation of the organelle membrane is significant, as the leakage of the cytosolic constituents into the extracellular space can initiate cell death by necrosis, which may trigger a powerful immune antitumoral response [100,122,123]. These cytosolic constituents in apoptosis will be sequestered by the intact membranes of apoptotic cells, which are phagocytosed by the infiltered macrophages [69,124]. To this effect, multiple animal studies have demonstrated the immune-inducing effects of PDT through immunogenic cell death (ICD) mechanisms, which ultimately damage tumor-associated vasculature, prevent metastasis, and enable tumor regression [103,125]. This ICD is activated through the extracellular release of specific signals (from tumor cells) known as DAMPs [108,110,126]. These DAMPs are characterized by adenosine triphosphate (ATP), calreticulin (CRT), heat shock protein 70/90 (HSP70/90) and group box-1 protein (HMGB-1), which are secreted or exposed on the extracellular membrane surface of the dying tumor cells [108,110,126]. These DAMPs are intracellularly expressed under normal conditions and translocated to the cell surface upon both ROS and ER stress [109,127]. These DAMPs proteins crucially mediate ICD through the activation of dendritic cells (DCs), which mature and subsequently present TAAs to cytotoxic T lymphocytes (CTLs), initiating tumor destruction in an antigen-dependent manner [107,125]. Interestingly, PDT using hypericin and other PSs could induce anti-tumor immune responses, following DAMPs releases. A recent report has shown that HYP-PDT could induce ICD through the surface expression of CRT (in dying cells), which under normal condition is expressed in the lumen of the ER [107,125]. Moreover, numerous reports demonstrated that the PDT anti-tumor immune response depends on the integrity of the tumor vasculature, infiltration of neutrophils into the tumor bed, and the secretions of pro-inflammatory signals into the tumor-draining lymph nodes (TDLN) [128,129]. This was supported by Henderson et al. (2004), showing that a high dose of PDT (128 J/cm^2^) could reduce anti-tumor immune responses by severely damaging tumor vasculature, thus preventing neutrophils infiltration to the tumor bed and releasing cytokines [130]. Conversely, Shams et al. (2015) showed that a low dose of PDT (48 J/cm^2^) could engender a persistent anti-tumor immune response by making the tumor vasculature porous enough to favor neutrophilic infiltration and a subsequent release of cytokines within TDLN to further recruit immune effector and regulatory T-cells [131]. Recently, Lamberti et al. (2019) demonstrated that methyl-aminolevulinic acid (Me-ALA)-PDT could induce anti-tumor immune responses [132] This ICD was mediated through a type I-interferon (IFN)-1-dependent pathway, which is able to activate DCs expressing co-stimulatory ligands (CD80, MHC-II), which eventually facilitated the priming of immune effector cells and tumor destruction in an antigen-dependent manner [132]. Recently, Yiao Jin et al. (2018) showed that 5-ALA-PDT was able to efficiently induce cervical cancer tumor destruction by reducing miR-34a and by increasing high-mobility group B-1 protein (HMGB1) (a nuclear protein that is excreted by damaged cells and binds to Toll-like receptor 4 (TLR4, which is predominantly expressed on macrophages and DCs) to activate T cell-mediated immune responses [133]. Corroborative findings on head and neck carcinoma patient serum post-PDT revealed an increase in HMGB-1, interleukin-6/10 (IL-6/10), which unfortunately correlated with a reduction in perforin, traducing the PDT immunomodulatory effects [134]. Interestingly, Naylor et al. (2006) and Li et al. (2010) were able to completely eradicate both local and lung tumor metastases on melanoma patients exposed to a combination of imiquimod (Toll-like receptor (TLR)-7 agonist) and indocyanine green (ICG) [135,136]. Similar findings were reported by Saji et al. (2006) and Wang et al. (2016) who successfully induced primary and metastatic tumor destruction of B16 murine melanoma tumors, following a combined therapy treatment involving intratumor injection of DCs or PDL-1 knockdown using siRNA, respectively [137,138]. Of late, Santos et al. (2018) have demonstrated the therapeutic efficacy of redaporfin–PDT synergism with anti-PD-1 immunotherapy in achieving the destruction of all visible head and neck tumors on a patient, who was previously exposed to surgery, radiotherapy, and multiple systemic treatments [64]. Lastly, He et al. (2016) used nanotechnology to demonstrate the synergistic and systemic anti-tumor immune effects of oxaliplatin chemotherapy and pyropheophorbide–PDT in inducing the destruction of primary treated and untreated colorectal tumor metastases, which is mediated by increased CRT and IFN-gamma producing CD8^+^ T cells [139]. Besides molecular oxygen and light, PDT mainly relies on PS’s passive accumulation in the diseased tissues to achieve its best therapeutic effect [140]. Unfortunately, PDT can adversely damage healthy tissues, when the PS non-specifically accumulates in the latter [73,141,142]. PDT efficacy can be affected by multiple factors including light accessibility to the tumor site and hypoxia [143,144,145]. To this effect, using multiple laser fibers, Jerjes et al. (2011) were able to successfully perform an ultrasound guided induced meso-tetrahydroxyphenyl chlorin (mTHPC) PDT on 100 patients having deep head and neck tumors [146]. This therapeutic strategy led to more than 50% objective response rate of which 5 patients became disease-free [146]. Likewise, Mallidi et al. (2016) were able to achieve significant tumor shrinkage on pancreatic adenocarcinoma patients by using computed tomography (CT)-guided PDT involving intratumoral insertion of optic fibers [147]. Therefore, with the aim to further enhance the therapeutic efficacy and reduce unspecific PS accumulation, novel strategies were developed by identifying clinically relevant TAAs serving as a molecular target for immunotherapeutic treatment using mAbs [148,149].

### 1.3. Melanoma Immunotherapy

#### 1.3.1. Introduction to Immunotherapy

Conventional cancer therapies (e.g., surgery, radiation, and chemotherapy) have shown limited therapeutic benefits in patients with metastatic disease [41,42]. Despite significant advances in the development of systemic therapies, the therapeutic usage of toxic agents remains a double-edged sword, potentially causing side effects and restricting treatment to certain therapeutic dosages [35,150]. Consequently, novel therapeutic strategies were developed to specifically treat patients with recalcitrant metastases. Cancer immunotherapy in the form of adoptive cell therapy (ACT) has shown the capacity to represent such a therapy, with the ability to harness the patient’s own immunity against tumors [99,100,101]. In order to achieve maximum therapeutic efficacy, cancer immunotherapy relies on antigen recognition of tumor cells by cells of the innate immune system such as DCs (antigen-presenting cells (APCs)), which subsequently migrate to secondary lymphoid tissue to prime CTLs that are able to destroy tumors in an antigen-dependent manner [49,54]. These ACT attributes led to the FDA approval of sipuleucel-T (in 2010), which is a DC vaccine that is used for the treatment of asymptomatic or minimally symptomatic castration-resistant prostate cancer [151,152]. Sipuleucel-T is able to activate autologous anti-tumor immune reactions toward prostate tumors overexpressing prostatic acid phosphatase tumor antigens [151,153,154]. This DC-based vaccine (sipuleucel-T) achieved high objective response rates in melanoma patients (8–15%), which were characterized by an improved overall survival (20%) mediated through a robust CTL and natural killer cell (NK)-dependent immune response [155,156,157].

Tumor-infiltrating lymphocytes (TILs) are another form of ACT introduced in 1988 to treat melanoma patients [41,54,158]. This therapeutic approach consists of isolating TILs from the patient’s tumor and expanding them ex vivo using interleukin-2 (IL-2) activation, before re-injection into the autologous patient to induce a CTL-dependent immune response [54,154,159]. This treatment was validated by multiple clinical trials confirming the overall objective responses of 49–72% with long-term remissions of >5 years in treated melanoma patients [108,109,110]. Efficacy was further enhanced when TIL was synergistically combined with conventional chemotherapy- and radiotherapy-induced lymphodepletion to selectively suppress regulatory T cells (Tregs) and other immunosuppressive cells in the tumor microenvironment [54,157,160]. Nevertheless, the clinical utility of this TIL therapy suffered major limitations due to restricted supply of the patient’s own tumor tissues, which can only be obtained by surgery. Moreover, economic viability might hamper its clinical application as it depends on highly trained medical staff who are able to expand isolated TIL cells after surgery in vitro [161]. Lastly, its therapeutic efficacy was only demonstrated on highly immunogenic melanoma and not on other malignancies [41,162]. Further improvement to overcome these hurdles led to the development of more sophisticated T-cell approaches which were exemplified by the most recent genetically engineered cells that successfully recognized the major histocompatibility complex-1 (MHC-1) negative tumors. This therapy helped patients replace the extracellular part of their cognate T-cell receptor by a TAA-specific recombinant antibody fragment, resulting in a chimeric antigen receptor (CAR) showing highly promising results [158,159,163,164]. Nowadays, CAR-T cells are additionally armed with transmembrane and intracellular co-stimulatory domains enabling natural T cell-activating functions (e.g., CD28 and 4-1BB) [41,156,165]. Between August and October 2017, Tisagenlecleucel, marketed as Kymriah (CD-19 specific CAR-T), gained clinical approval to treat B-cell acute lymphoblastic leukemia (ALL), chronic lymphocytic leukemia (CLL), non-Hodgkin lymphoma (NHL), and primary mediastinal B-cell lymphoma (PMBCL) [163]. Yet, this therapeutic success has only been limited to hematological malignancies as a result of CTL long-term persistence, conferring long lasting anti-tumor immune effects [159]. On the other hand, CAR-T cells have been inefficient in treating solid tumors such as melanoma due to multiple factors including tumor expression of programmed death ligand-1 (PD-L1: causing T-cell exhaustion), extracellular release of inhibitory cytokines (e.g., CXCL1, CXCL5, and CXCL12), hypoxic microenvironments, and distorted tumor vasculature, which impairs immune functions [159,166]. To mitigate these effects, targeted therapies using mAbs were developed, which revolutionized cancer treatment, due to advanced knowledge gained from molecular biology and tumor immunology mechanisms regulating cancer progression. These advances led to the establishment of immunotherapeutic treatments involving the production and commercialization of mAbs, which exert their therapeutic effects upon binding to tumor cells expressing cognate antigens. Traditionally, these immunotherapeutic treatments exercise their cytotoxic effects through ADCC, complement-dependent cytotoxicity (CDC), or receptor blockade [31,167,168,169]. However, the successes of these mAbs based on immunotherapeutic treatments rely on high dosages and the multiple use of therapeutic agents [11,170,171]. In 1998, IL-2 was FDA-approved as an immunomodulating treatment (activating anti-tumor immune responses) of metastatic melanoma patients, which was due to its ability to mediate durable objective responses [11,171]. Despite its good initial response, its widespread application was restricted due to patient relapses from the treatment and the requirement of high therapeutic dosages causing significant toxicity [11]. Hence, further refinement of immunotherapeutic treatments was performed to achieve clinical fruition.

#### 1.3.2. Antibody-Based Immunotherapy

**Figure 3:**
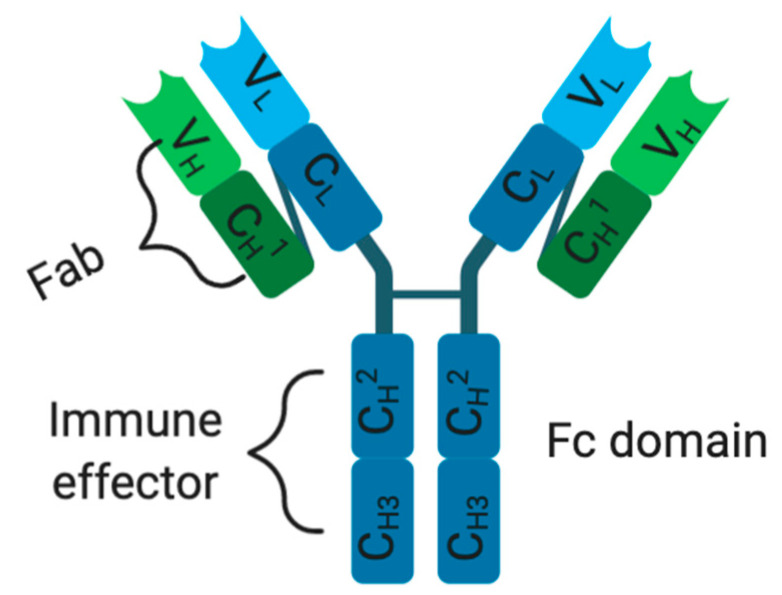
Monoclonal antibody structure. C: constant domain, V: variable domain, H and L: heavy and light chains.

In order to overcome ACT drawbacks, immunotherapeutic treatments were developed in a form of molecular-targeted therapies using mAbs. However, each mAb possesses an antigen-binding region known as a fragment variable region (Fab) and a constant region (fragment crystallizable: Fc domain) with an effector function (Figure 3). The Fv (fragment variable region) fragment of a mAb is made up of a variable light chain (V_L_) and a heavy chain (V_H_), containing three complementarity-determining regions (CDRs) and four framework regions (FRs) [168,172,173]. Traditionally, these mAbs exercise their cytotoxic effects through their Fc domain, within the constant region, which functions by interacting with immune effector cells and mediates tumor destruction via ADCC, CDC, or receptor blockade (Figure 3) [167,168,174,175]. Unfortunately, the success of this immunotherapeutic treatment (e.g., high-dose-IL-2) relies on high dosages and multiple treatment schedules, thus limiting clinical benefits [11,155,176,177,178]. Therefore, ipilimumab, a fully human mAb (immunoglobulin G1, or IgG1) that targets and blocks CTLA-4, was developed and clinically approved by the FDA as the first immune checkpoint inhibitor to treat metastatic melanoma patients [148,179,180]. CTLA-4 is a CD28 homolog, a T-lymphocyte co-stimulatory receptor that normally binds to cognate B7-ligand expressed on APCs such as DCs to activate T cell function [177,179,180]. Unfortunately, when CTLA-4 outcompetes CD28 for binding on a cognate B7 ligand, as a result of higher affinity and avidity, it activates T cells exhaustion, compromising antitumor immune responses [177,179,180]. Hence, by preventing CTLA-4 interaction with B7, ipilimumab acts to reinvigorate previously exhausted T-cells and boosts antitumor immunity through enhanced immune effector functions [177,179,180]. This therapeutic success (CTLA-4) spurred further development, leading to the FDA approval (in 2015) of new immune cell blockade (ICB) anti-PD-1 mAbs (pembrolizumab and nivolumab) binding respectively to their natural programmed death ligands 1/2 (PDL-1 and PDL-2) largely expressed on various immune cells (T cells, B cells, NK cells, macrophages, and DCs) and tumor cells [181,182]. For instance, nivolumab gained clinical approval following the Check-Mate006 clinical trials on patients with unresected and advanced melanoma [181]. During this study, nivolumab was shown to produce a progression-free survival (5.1 versus 2.2 months) and an objective response rate (40% versus 13.9%) superior to the DTIC-treated patients, respectively [181,183]. Similarly, pembrolizumab showed better therapeutic benefits, which were characterized by higher progression-free survival (e.g., 6 months in 47.3% of biweekly treated patients) and overall survival (e.g., 1 year survival in 74% biweekly treated patients) when compared to ipilimumab (e.g., 6 months in 26.5% and 58% of overall survival) [181,184]. In spite of their therapeutic successes (anti-CTLA-4 and anti-PD-1), only a subset of patients manifests a durable response [180,185]. Currently, a palliative approach is being tested in a phase III clinical trial (NCT02224781) combining ICB therapies (ipilimumab and nivolumab) with dabrafenib and trametinib (NCT02224781). This combinatorial approach was supporting Sanlorenzo et al. (2018) findings, showing how BRAFi/MEKi treatment could be synergized with anti-PD-1 therapy to kill BRAF^V600E^-positive melanoma tumor cells [186]. Another phase II clinical trial (NCT02908672) investigating the combination of atezolizumab (fully humanized anti-PDL-1) with cobimetinib (MEK inhibitor) and vemurafenib against vemurafenib and cobimetinib treatment is presently being tested on metastatic melanoma patients. Lately, a phase I clinical trial (NCT02967692) was designed to assess the safety and efficacy of the spartalizumab (anti-PD-1 mAb) combination with a BRAF inhibitor (dabrafenib) and an MEK inhibitor (trametinib) in unresectable or metastatic BRAF^V600E^ mutants. The success of this antibody-based immunotherapeutic treatment has been limited by multiple factors: (1) non-specific biomarker selection leading to the identification of irrelevant TAAs, (2) inefficacy of mAbs to treat cancers, (3) reduced mAbs internalization into tumor tissues (due to their bulky size), (4) production of neutralizing antibodies (or anti-idiotypic antibody) against mAbs of human origin, (5) off-target effects and immunogenicity when used in humans with functional immune systems, limiting repeated treatment dosage schedules and (6) common sides effects such as fatigue, rash, skin disorders, endocrinopathies, diarrhea, pneumonitis, and colitis [187,188,189,190].

As such, despite early promise, the clinical application of therapeutic murine mAbs was severely hampered by their incapacity to efficiently activate human effector functions and their immunogenicity, which gave rise to human anti-mouse antibodies (HAMA) [187]. This immunogenic response toward the fully xenogeneic murine mAb led to poor therapeutic efficacy due to the neutralization and/or premature clearance of mAbs from the bloodstream, causing serious life-threatening side effects such as allergic and immune-mediated reactions (e.g., thyroiditis) [191,192]. This therapeutic inefficacy was demonstrated when cutaneous T-cell lymphoma (CTCL) and melanoma patients were treated with murine mAbs T101 and 9.2.27, respectively [193]. Half of the treated CTCL patients and three melanoma patients were shown to develop immunogenicity, which was characterized by an increased production of human antibodies against mouse immunoglobulin G (mIgG), especially with a repeated treatment cycle [193,194]. This immunogenic response is a significant problem, as it markedly compromised the widespread and repeated application of mAbs to treat various diseases. To mitigate these effects, recombinant protein technology was developed and led to the engineering of chimeric antibodies with ameliorated therapeutic outcomes [35]. These antibody formats consist of fusing an antigen-binding region (Fab: endowed with the antigen-binding capacity of mouse xenogeneic origin) to a human antibody Fc region possessing the effector functions that mediate ADCC [195]. These chimeric antibodies have very low levels of immunogenicity, enabling repeated dose treatment schedules with conserved efficacy of the parental mAb [168]. Rituximab (FDA-approved in 1997) is an example of a chimeric anti-CD20 mAb (consisting of a murine CD20 binding variable region of IgG1 mAb IDEC-2B8, which is genetically fused to a human IgG1 and kappa constant regions) used to treat multiple cancers [196]. Rituximab was shown to moderately improve therapeutic efficacy when treating melanoma patients [197]. In contrast, Velter et al. (2014) demonstrated that rituximab could worsen melanoma treatment or induce melanoma while treating B-cell lymphoma patients [198]. These results prompted the further optimization of mAbs, aiming at improving chimeric antibody properties by humanizing the fragment variable regions (Fab), which possess antigen-binding activity. Humanization of an antibody can be performed through various methods including the grafting of CDRs, veneering through surface manipulation of the framework region (FR) and transgenic mice using hybridoma technology. During the grafting method, xenogeneic V_H_ and V_L_ of the variable region sequence (CDRs) are joined to the human depleted CDR immunoglobulin scaffold [196,197]. Although this process drastically reduces the antigenicity of murine mAb in humans, it may alter the humanized antibody–antigen binding capacities, which in turn can influence its pharmacokinetic properties. The further improvement of mAbs can be achieved through the veneering method, which minimizes xenogeneic mAb antigenicity in human, by substituting xenogeneic FR-exposed residues with those mostly found in human antibodies. This is particularly relevant, as antigen-binding affinity relies heavily on the topography and chemical structure of the CDRs and some framework residues to maintain its binding affinity [199,200,201,202]. This was confirmed by Padlan (1991), who reported that human and rodent-derived immunoglobulin V_H_ and V_L_ possess unique features in exposed residues, which vary across the species [203,204]. Hence, an ideal antibody humanization should generate a product with (1) reduced immunogenicity and (2) conserved antigen-binding affinity on the non-human CDRs. To achieve these goals, humanization procedures should substitute exposed residues within the FR regions of the human scaffold with the murine exposed residues. This can be performed by selecting the human Fab region showing the greatest sequence homology to the specific murine Fab region consensus sequence [205]. Yet, few studies were able to simultaneously preserve the antigen-binding properties and reduce the murine-derived CDR-induced antigenicity by simply grafting the latter xenogeneic CDR to the human-depleted immunoglobulin [205]. These limitations paved the way to the development of transgenic mice, which enabled the production of a fully human antibody. These mice were engineered to possess functional human immunoglobulin transgenes, replacing their mouse orthologues, which are genetically inactivated [206,207]. In 1998, a humanized mAb gained FDA approval to treat human epidermal growth factor receptor 2 (HER2)-positive breast tumors [196,197,203,206]. Thereafter, ipilimumab (targeting CTLA-4, FDA-approved in 2011) and spartalizumab (humanized IgG4-PD-1) were developed to treat melanoma patients [208,209]. Indeed, while the clinical efficacy of DTIC in metastatic melanoma was low and did not offer any confirmed survival benefit, alternative treatment guidelines (based on the use of fully human mAbs) were being approved by the FDA [210]. For instance, patients with unresectable (advanced) stage III or IV melanoma received ipilimumab and nivolumab (targeting PD-1) as concurrent therapy, resulting in a 3-years overall survival rate of 63% in 94 patients [211]. As a result of the above-mentioned clinical successes, it became obvious that mAbs could be used as immunotherapeutic agents. Recently, antibody genetic engineering has permitted the production of genetically truncated versions, which are devoid of their effector Fc domain. These unnatural antibodies still retain their antigen-binding properties and can be generated through the randomization of CDRs of the Fab regions (Figure 3) [107,212]. Interestingly, these new antibody formats can be genetically or chemically fused to a fusion protein or cytotoxic agent to exert their potent effects as previously reported [197,208,213,214]. For example, a single-chain fragment (scFv) consisting of V_H_ and V_L_ chains of a mAb (about 30 kDa) linked by a short peptide sequence can be genetically fused using interdomain chains to form multivalent antibodies, such as diabody (60 kDa) or triabody (90 kDa), resulting in high-avidity properties [173,200]. However, the therapeutic activity of these novel antibodies will solely depend on the function of their conjugated warhead toxin or toxic agents. Based on these observations, it was quickly realized that naked mAbs against TAAs would not reach therapeutic fruition in existing pre-clinical animal models and that it would likely need to be coupled with toxic agents (e.g., small molecule toxins or PSs) to achieve improved anti-tumor responses.

#### 1.3.3. Overcoming Monoclonal Antibody Limitations Using Antibody-Drug Conjugates

**Figure 4:**
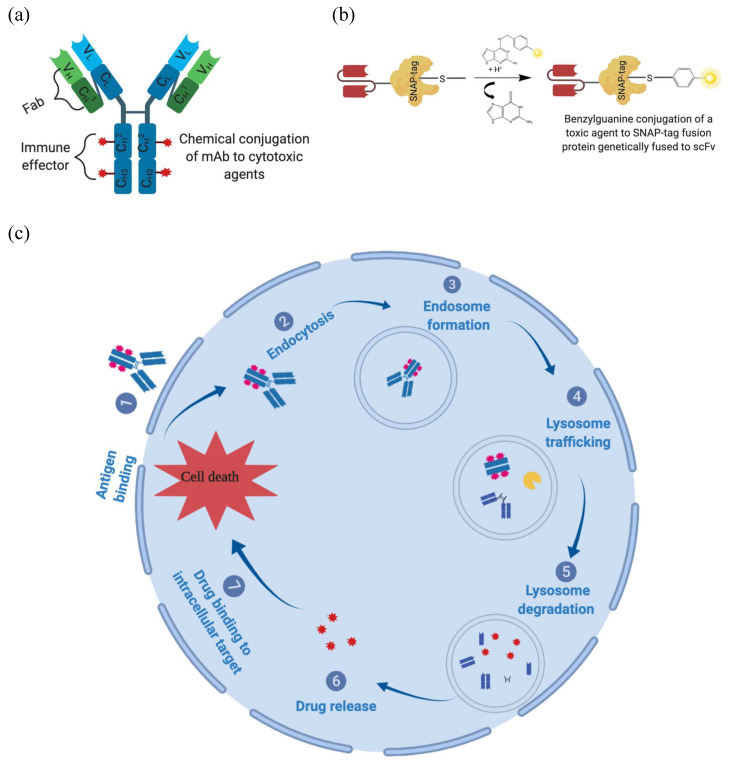
A summary of the targeted immunotherapy using monoclonal antibody–drug conjugates and SNAP-tag fusion proteins. (**a**) Monoclonal antibodies (mAbs) or immunoglobulin (IgG) constitute of a fragment of variable (V) and constant (C) region domains chemically conjugated to a cytotoxic agent, and (**b**) a single-chain variable fragment (scFv) genetically fused to SNAP-tag fusion protein and conjugated to a benzylguanine modified cytotoxic agent. (**c**) Mechanism of action of both antibody–drug conjugate (ADC) types.

ADCs consist of chemically or enzymatically conjugated mAbs to a toxic warhead that is specifically delivered into targeted tumor cells overexpressing cognate TAAs. ADC-mediated cytotoxicity begins with immunoconjugate binding to the TAA receptor subsequently inducing internalization as an ADC–antigen complex within the targeted cell through receptor-mediated endocytosis, forming ADC-loaded endosomes trafficked to intracellular compartments including lysosomes (Figure 4). Once in the lysosomes, the payload is released either through an enzymatic digestion or a pH-induced degradation (low pH) of the linker or mAb backbone, causing the cytosolic release of cytotoxic drugs to reach their intracellular targets and induce cell death (Figure 4). ADCs were conventionally generated through the chemical conjugation (alkylation or acetylation) of lysine or reduced inter-chain disulfide residues of mAbs to cytotoxic payloads [215,216,217]. Unfortunately, these methods usually produced heterogeneous products composed of a mixed drug-to-antibody ratio (DAR) with different pharmacokinetic efficacies and safety profiles [209,216,218]. For instance, a high DAR of 8 drug molecules conjugated per antibody was associated with ADC deterioration, increased premature clearance from bloodstream circulation, reduced stability under stress conditions, and an increased ability to aggregate and cause an immunogenic reaction [219,220,221,222]. On the other hand, a DAR of 4:1 had been optimized with increased serum half-life [217,223,224]. Moreover, ADCs were improved through protein engineering. These improvements were made by conjugating an antibody to the cytotoxic payload using a linker molecule. This was achieved through an enzymatic reaction or the insertion of a synthetic reactive cysteine functional group within the antibody protein sequence [31,223,224]. However, the critical selection of the linker influenced the successful design of an ADC delivery system. A linker plays an important role, because it ensures the covalent linkage of mAbs to the effector molecule. This covalent linkage can also impact ADC stability by generating different DARs [218,225]. This is crucial, as highly potent anti-mitotic toxin molecules such as monomethyl auristatin-F (MMAF) could be attached to a linker and result in improved stability in the bloodstream. This is to ensure that the delivery of the cytotoxic payload within the targeted cell is specific while preventing its premature release, which may cause severe side effects [209,218,226]. In order to reduce the heterogeneity of the conjugation products, fusion protein conjugation methods were ameliorated. These fusion proteins consisted of genetically fusing mAbs or antibody fragments (e.g., scFv) with protein gene tags [25,227,228,229]. These protein tags possess a self-labeling activity, providing a unique site of conjugation that generates optimal stoichiometric ratios of antibody to cytotoxic payloads, thus creating homogeneous conjugates. This is an advantageous coupling strategy when compared to traditional methods producing heterogeneous conjugate mixtures with different pharmacokinetic behaviors. The protein tag enables the fusion protein to specifically react with a chemically modified cytotoxic payload, which avoids chemical conjugation while maintaining product homogeneity [227,228,229,230,231]. For example, CLIP-tag and Halo-tag fusion proteins react with benzylcytosine (BC) and chloroalkane-modified molecules, respectively. Tetracysteine-tag and SNAP-tag react respectively with biarsenical (e.g., fluorescein arsenical hairpin (FLAsH) or resorufin arsenical hairpin (ReAsH)) and benzylguanine (BG)-modified compounds [228,232,233,234].

SNAP-tag is a suicidal enzyme, resulting from an engineered version of the 20 kDa human DNA repair protein known as O_6_-alkylguanine-DNA-alkyltransferase (AGT), which can specifically and rapidly react with BG derivatives (Figure 4B). SNAP-tag fusion proteins catalytically react better (50-fold) with BG-modified compounds when compared to AGT, which under normal conditions remove alkyl adducts from the O^6^ and the O^4^ positions of guanine and thymine to protect cells from the potent effects of alkylating agents [229,235,236]. SNAP-tag is a simple conjugation method combining the following: (1) specificity of conjugation (reacts only with BG-modified substrates), (2) shorter conjugation reaction (30 min for BG fluorochromes and 2 h for cytotoxic payloads), (3) flexibility of the expression system (bacteria, yeast, or mammalian) and availability of various BG-modified substrates, (4) no reactivity with other cellular substrates, (5) no requirement for activating substrates for the conjugation reaction, and (6) a 1:1 stoichiometric reaction generating homogeneous products by only reacting with BG molecules [167,237,238,239,240,241]. These ADCs are a novel alternative that may alleviate the side effects and low intracellular accumulation of toxic payloads associated with chemotherapy (DTIC) and PDT in melanoma treatment.

#### 1.3.4. Recombinant Antibody-Drug Conjugates for Melanoma Treatment

To address the limitations associated with conventional therapies and naked mAb-based immunotherapy, recombinant ADCs were developed in the form of scFv–SNAP-tag fusion proteins conjugated to MMAF endowed with the ability to specifically detect and kill melanoma and other cancer cells overexpressing EGFR using nano and picomolar concentrations [242,243]. The efficacy of these MMAF ADCs was confirmed on melanoma cells that overexpress multiple receptors such as melanotransferrin and the epidermal growth factor receptor 3 (HER3) [1,16,33]. Since the therapeutic effect of these ADCs depends on their toxic warheads, it becomes pertinent to discuss how these drugs achieve their cytotoxic effects.

**Figure 5:**
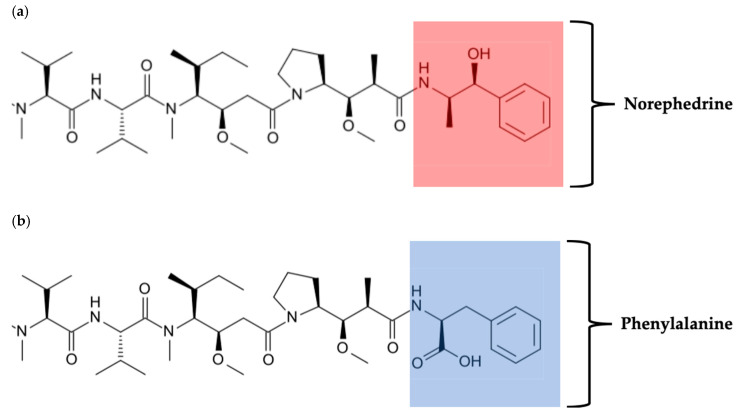
Monomethyl Auristatin E and F structures. Amino acid components of monomethyl auristatin E (MMAE) and monomethyl auristatin F (MMAF). (**a**) The fifth amino acid of MMAE and (**b**) MMAF are labeled and highlighted in color. The difference in the carboxyl terminal between the norephedrine group of MMAE (pink) and the phenylalanine MMAF (blue) is highlighted, respectively.

Monomethyl Auristatin E/F (MMAE or MMAF (AURIF): Figure 5) are equipotent, anti-mitotic, cytotoxic drugs, which are structurally derived from Dolastatin-10, a novel pentapeptide agent found in the marine mollusk *Dolabella auricularia* [244,245,246,247]. MMAE/F exert their anti-tumor activity by interacting with the vinca alkaloid-binding site of tubulin, thereby inhibiting tubulin polymerization, disrupting microtubule assembly, and activating a G2/M cell cycle phase arrest, leading to cell death [244,245,248]. To date, both MMAE and MMAF are used as cytotoxic warheads in various ADC types, due to the desirable specific toxicity associated with MMAE and dolastatin-10 in clinical trials [245,246,247,249]. In this regard, several auristatin-based ADCs were generated, of which brentuximab vedotin (anti-CD30 linked to MMAE) was one such ADC, which gained FDA approval in August 2011 to treat CD30-positive Hodgkin’s lymphoma patients [17,222,248,249]. Nevertheless, MMAE (cell membrane permeable) was shown to be more cytotoxic as a free drug as compared to MMAF (cell membrane impermeable) due to cell membrane permeability differences [244,250,251,252]. MMAE has been shown to potentially induce a bystander effect upon release from the antibody. This effect can either be advantageous when MMAE passively diffuses into nearby tumor cells or negative and detrimental when MMAE accumulates within normal cells, thereby causing serious side effects [250,253]. Conversely to MMAE, ADC–MMAF has shown to only release potent MMAF bound to the cysteine residue via a non-cleavable linker, post internalization via endocytosis and cytosolic release, thus preventing any off-target cytotoxicity [17,215,251]. This non-bystander effect was observed because MMAF possesses a charged C-terminal phenylalanine residue, which prevents cell membrane passive diffusion, thus attenuating its unspecific cytotoxic activity [33,245]. Recently, HER2-conjugated MMAE/F was shown to have superior potency on gastric, pancreatic, and other cancers that overexpress HER2 when compared to trastuzumab [16,244,253]. Moreover, using scFv-SNAP-tag fusion proteins, MMAF was shown to specifically detect and kill breast and skin cancer cells expressing EGFR, while sparing antigen-negative cells [242,243]. The potency of these MMAF-based ADCs was confirmed on melanoma cells that overexpress the receptor tyrosine-protein kinase erbB-3, which is also known as HER3 (HER3 allows the escape of tumor cells from vemurafenib-targeted treatment of BRAF-positive melanoma cells) [1,16,33]. Interestingly, this ADC–MMAF was highly toxic to melanoma cells, irrespective of their BRAF status and showed superior cytotoxic activity to the BRAF inhibitor vemurafenib in preventing melanoma. In line with this, Smith et al. (2006) successfully killed p97-expressing melanoma cells by conjugating L49 mAb to MMAF (L49–vcMMAF) [33]. This targeted approach was shown to be more effective in killing melanoma cells highly expressing p97 (280,000 in melanoma versus 80,000 sites per cell on healthy tissues) and was shown to be associated with strong immunochemical staining of p97, which is present in 62% of metastatic melanoma tumor biopsies [33]. This achievement was preceded by studies conducted by Siemers et al. (1997) and McDonagh et al. (2003), who showed that the recombinant fusion protein L49–scFv-bL (containing the antibody-binding region of L49 fused to *Enterobacter cloacae* r2-1β-lactamase (bL)) was able to cure a melanoma tumor and other cancers in nude mice when combined with well-tolerated doses of 7-(4-carboxybutanamido) cephalosporin mustard prodrug (CCM) [218,254]. Moreover, this recombinant fusion protein L49-scFv-bL had better therapeutic outcomes than the chemically conjugated L49-Fab-bL (monoclonal antibody fused to bL by heterobifunctional cross-linking reagent) due to improved DAR and thus enabling it to cure renal cell carcinoma with a lower dose of CCM as opposed to L49-Fab-bL, which required maximal tolerated CCM doses to produce the same effect [254]. In summary, using ADCs as a targeted approach becomes crucial and greatly contributes to the precision medicine treatment of therapy-resistant melanoma.

### 1.4. Photoimmunotherapy

Ideally, the most efficient cancer therapy is one that can simultaneously cause tumor destruction by virtue of the toxic agent used and the ability of the latter to induce an anti-tumor immune response toward both primary and metastatic tumors [126,132]. With this in mind, PIT was developed as a light-dependent targeted cancer therapy, using antibody photoconjugates (APCs: functionalizing an mAb to a PS to form a phototoxic immunoconjugate) to selectively and spatially deplete irradiated tumor cells [36,131,255]. PIT was originally proposed as a novel therapeutic treatment following the observation that it was able to kill illuminated cancer cells membrane-bound by mAb (trastuzumab and panitumumab) conjugates to near-infrared dyes (NIR) such as 700DX (IR700), which by producing a death-inducing amount of ROS upon excitation achieves the following: (1) compromises cell membrane integrity, (2) causing water influx into the cell, which eventually results in (3) cell blebbing/lysis causing tumor destruction [35]. Indeed, first pioneered by Mitsunaga et al. (2011), PIT mainly involves the use of the NIR phthalocyanine dye IR700 [256]. Excitation of IR700 with NIR light at approximately 690 nm allows penetration of at least several centimeters into tissues [257]. On this basis, fiber-coupled laser diodes with diffuser tips, which have previously been used in PDT to treat brain tumors and peritoneal metastasis of ovarian cancers, can also be applied in PIT to ensure the delivery of NIR light within several centimeters of deeply rooted and otherwise inaccessible tumors [256,258]. The therapeutic efficacy of PIT has been demonstrated in multiple pre-clinical animal models of human cancers including breast (HER2), pancreatic (carcinoembryonic antigen), bladder (EGFR), prostate (prostate-specific membrane antigen), glioblastoma (mesothelin and EGFR), and melanoma (EGFR) [17,37,39,41,259,260,261,262,263]. PIT offers several advantages compared to standard ADCs by: (1) only affecting irradiated tumor cells, (2) producing no off-target effects with unbound APCs remaining nontoxic (as opposed to ADCs that inherently come with side effects limiting therapeutic dosages), (3) by only requiring cell surface attachment to the cognate antigen to exert its potency on tumor cells (while ADCs need binding to TAA, internalization, and release of the toxic warhead once in the cell), (4) by being water-soluble as opposed to the most potent ADCs, which are mostly hydrophobic and incompatible with an mAbs buffer system [35]. So far, monotherapy using PIT has failed to simultaneously control primary and metastatic tumors. This therapeutic failure was associated to the development of immune resistant mechanisms, involving the expression of checkpoint inhibitors such as PD-1 [131,255]. To overcome this obstacle (immune resistance), combination therapy consisting of immune cell blockade (ICB) and PIT was developed [255]. According to Nagaya et al. (2019), CD44-targeting PIT could synergize with PD-1 ICB therapy to completely reject both primary irradiated and non-irradiated distant CD44-positive MC38 tumor metastases in a systemic immune response known as the abscopal effect [255]. The efficacy of this combined therapy was based on the ICB (anti-PD-1) ability to reverse immune resistance through the reinvigoration of pre-existing exhausted tumor antigen-specific T cell responses, thus establishing a systemic immune memory that is capable of clearing tumors when re-challenged [255]. Moreover, PIT has the advantage of being specific by inducing cell death only in irradiated target-expressing cancer cells, while sparing adjacent non-targeted expressing cells [35,36,256,264]. This was exemplified by Von Felbert et al. (2016) and Amoury et al. (2016) demonstrating the specific phototoxic effect of SNAP-tag based PIT using the NIR PS IR700, which specifically enabled the diagnosis and tumor destruction of melanoma and breast cancer cells, respectively overexpressing EGFR, CSPG4, and epithelial cell adhesion molecule (EpCAM) receptors, using sub-nanomolar IC_50_ concentrations (32–165 nM) [17,36]. As such, despite highly speculative, PIT may (in the near future) be used as a neoadjuvant therapeutic treatment to specifically and efficiently eradicate melanoma, since it is currently being evaluated in the third phase of a clinical trial (NCT03769506) for the treatment of recurrent and advanced head and neck squamous cell carcinoma (in patients who have failed or progressed on or after at least 2 lines of traditional cancer therapy) using cetuximab-IR700 [35].

## 2. Conclusions

For many years, melanoma was thought to be resistant to conventional therapies and particularly to PDT. Bypassing this PDT resistance was achievable, even in highly pigmented melanoma, by using interventions that can temporally reduce the melanin pigment optical interference or through the usage of NIR PS absorbing in the 700–900 nm spectral region. The efficacy of PIT is noteworthy in inducing cell death in specifically irradiated target-expressing cancer cells only while sparing adjacent non-targeted expressing cells. With significant research efforts, targeted therapies were developed and concomitantly improved PS delivery into targeted tumor cells while lowering toxic agent concentrations and increasing therapeutic efficacy. These therapies include mAbs, which had limitations due to their size, leading to the use of scFvs (the smallest functional unit of an antibody). Single-chain fragments of variable regions (scFvs) represent promising antibody formats with beneficial clinical utility, owing to their small size, facilitating conjugate penetration within tumor tissues as well as their advantageous pharmacokinetic behavior (e.g., rapid clearance from circulation) and low production costs compared to mAbs. Furthermore, these scFv fragments can be genetically assembled into multivalent conjugates with increased serum circulation and equipped with the ability to simultaneously recognize numerous targets. These formats have especially been used in the generation of recombinant ADCs bearing SNAP-tag, which exhibit a 1:1 stoichiometric reaction (thus generating homogeneous products by reaction with BG molecules) and can overcome the limitations of traditional ADCs which have a DAR > 4. Additionally, it was recently realized that targeted therapy in the form of PIT could induce host immune responses, which when combined with the known immunogenic potential of melanomas and immune cell blockade could activate systemic anti-tumor immune responses (abscopal effect) that may lead to clinical success in the management of advanced metastatic melanomas.
